# Fabrication of quantum dot and ring arrays by direct laser interference patterning for nanophotonics

**DOI:** 10.1515/nanoph-2022-0584

**Published:** 2023-01-10

**Authors:** Yun-Ran Wang, Im Sik Han, Mark Hopkinson

**Affiliations:** Department of Electronic and Electrical Engineering, The University of Sheffield, Sir Frederick Mappin Building, Sheffield S1 3JD, UK

**Keywords:** direct laser interference patterning, droplet epitaxy, molecular beam epitaxy, quantum dots, quantum rings

## Abstract

Epitaxially grown semiconductor quantum dots (QDs) and quantum rings (QRs) have been demonstrated to be excellent sources of single photons and entangled photon pairs enabling applications within quantum photonics. The emerging field of QD-based nanophotonics requires the deterministic integration of single or multiple QD structures into photonic architectures. However, the natural inhomogeneity and spatial randomness of self-assembled QDs limit their potential, and the reliable formation of homogeneous and ordered QDs during epitaxy still presents a challenge. Here, we demonstrate the fabrication of regular arrays of single III–V QDs and QRs using molecular beam epitaxy assisted by *in situ* direct laser interference patterning. Both droplet epitaxy (DE) GaAs/AlGaAs QDs and QRs and Stranski–Krastanov (SK) InAs/GaAs QDs are presented. The resulting QD structures exhibit high uniformity and good optical quality, in which a record-narrow photoluminescence linewidth of ∼17 meV from patterned GaAs QD arrays is achieved. Such QD and QR arrays fabricated through this novel optical technique constitute a next-generation platform for functional nanophotonic devices and act as useful building blocks for the future quantum revolution.

## 1 Introduction

The quantisation of energy levels in low-dimensional semiconductor nanostructures, particularly quantum dots, offers intriguing optical and electronic characteristics, which endow them with great potential in the fields of optoelectronics, nanophotonics and quantum technologies. As ideal solid-state single photon and entangled photon sources, semiconductor self-assembled QDs and QRs have enabled a wealth of new physics and applications such as solid-state quantum emitters and qubit gates for quantum computing [1–3]. Many of these applications require the incorporation of single QDs or regular arrays of QDs within nanophotonic structures such as photonic crystal cavities [4, 5], micropillar [6, 7] and microdisk cavities [8] to allow efficient coupling between the optical mode and the embedded QD structures. The ability to control the light–matter interaction strength in integrated photonic structures enables a wide range of cavity quantum electrodynamics such as Purcell enhancement and single-photon nonlinearity [9]. Nevertheless, most of this work to date has been performed on locating randomly positioned self-assembled QDs, usually by carefully selecting an individual dot from a large number of candidates. Whilst this may be an acceptable approach for physical investigations, it would lead to a very low yield for scalable fabrication. The capability to realise scalable and deterministic fabrication of single QD nanostructures that are spatially ordered and with identical quantum states and characteristics would constitute a key step towards future functional device applications.

Many excellent research works have been carried out in the past years to precisely control semiconductor QD structures with respect to size, density and position. Site-controlled growth of QDs using ex situ lithographic techniques such as electron beam lithography [10–12] and nanoimprint lithography [13–15] present a viable approach; nevertheless, these involve complex fabrication processes and the resulting properties of the structures have not, in general, matched those of random self-assembled nanostructures. Therefore, an alternative nanofabrication paradigm that can both maintain the high crystalline and optical quality of materials through bottom-up natural epitaxial self-assembly, but also allows top-down lithographic positioning would be highly attractive. This would be especially true if it can be performed without the need for multi-step processing in which the introduction of impurities in the epitaxial regrowth step is hard to suppress.

Direct laser interference patterning (DLIP) has been demonstrated to be a powerful approach for fabricating large-area periodic micro- and nano-scale structures, with many advantages over conventional lithographic methods. It is a mask-less approach that can be applied over a large area in a single step and is therefore cost-effective. Laser surface modification is dependent on the local interaction between the optical field and the material and can include photothermal, photochemical or photomechanical mechanisms [16, 17]. Various surface structures such as arrays of gratings, holes or pillars have been fabricated on a variety of materials, including polymers [18], metals [19] and ceramics [20]. Many of these structures are formed by laser ablation or deformation processes using relatively high-energy pulsed lasers. However, the modification of surfaces can take place well below these energy thresholds. Recently, research has shown the ability to pattern semiconductor surfaces by direct laser writing [21], to induce the formation of nanostructures by direct laser irradiation [22], and to arrange semiconductor nanostructures in a controlled manner using DLIP within a molecular beam epitaxy (MBE) chamber, including the formation of InGaAs QD arrays [23, 24]. The significance of these works is that they show that near-surface absorption of nanosecond ultraviolet (UV) pulses of moderate laser energy is sufficient to induce surface structuring during epitaxy, i.e. concurrently with materials formation within the same vacuum environment. However, in these early works, precisely controlled single dot arrays were not achieved and associated optical properties were never demonstrated. Furthermore, the fabrication of droplet epitaxy (DE) QD and QR arrays using DLIP has not been reported.

In this work, we report on the fabrication of ordered arrays of high-quality III–V QD structures by combining the advantages of MBE self-assembly and the simplicity of *in situ* DLIP. A detailed investigation of the structural and optical properties of ensemble DE GaAs/AlGaAs QD and QR arrays and SK InAs/GaAs QD arrays is presented. By optimising the growth and DLIP conditions, we could achieve uniform arrays of single QDs and QRs. The optical properties of such QD arrays revealed by the low-temperature photoluminescence (PL) measurements indicate good size homogeneity and optical quality. This *in situ* technique paves the way for the fabrication of single QD arrays and enables their practical integration into photonic device platforms.

## 2 Experimental details

### 2.1 DLIP-MBE setup

**Figure 1:**
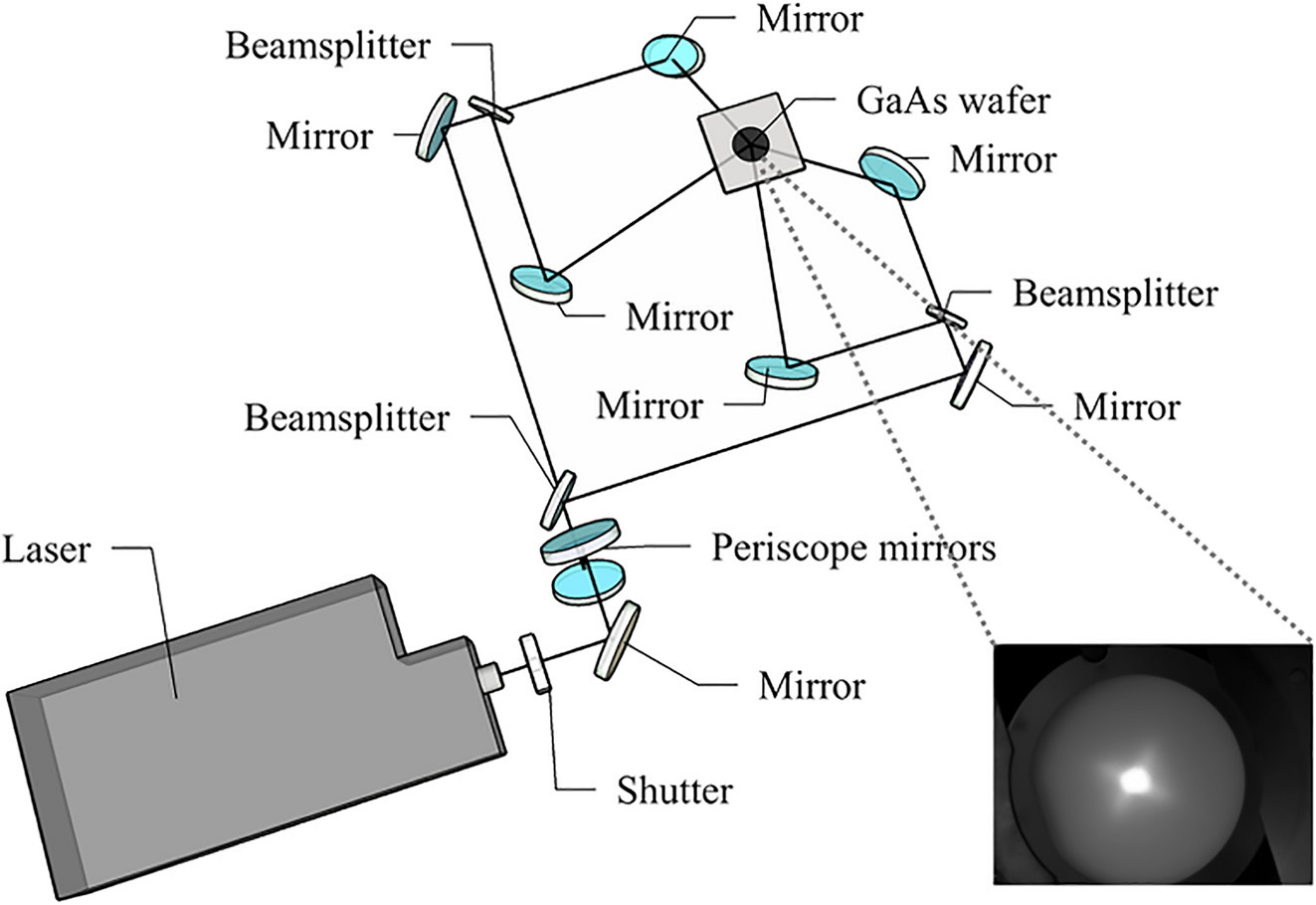
Schematic configuration of the four-beam DLIP-MBE setup. The insect shows a CMOS camera image of the four superimposed beams on an InGaN wafer.

The MBE chamber (MBE-Komponenten GmbH, Octoplus 400) is equipped with symmetric anti-reflective optical viewports that allow four coherent laser beams to converge on the growing wafer. An experimental setup to produce four-beam laser interference patterns in the MBE system with a controlled incidence angle was designed, as depicted in Figure 1. A flash-lamp pumped Nd:YAG laser (InnoLas SpitLight 1000) with a wavelength of 355 nm, *p*-polarisation, a pulse duration of 7 ns, a pulse repetition rate of 5 Hz, and a beam diameter of 5 mm was utilised. An external shutter was used to obtain single pulse exposure from the 5 Hz repetitive publishing. In the setup, the output laser beam was split into four sub-beams with identical optical paths using three 50:50 UV dichroic beam splitters. After that, these four sub-beams were reflected by four symmetrically placed UV mirrors with equivalent azimuthal angles of 0°, 90°, 180° and 270°, and then recombined on the centre of the 2-inch sample surface at an incidence angle of 58°. The UV beams were viewed from the luminescence of an InGaN wafer, which allows us to observe the image of the beam spots with an upward-facing CMOS camera, as shown in the inset of Figure 1. This optical arrangement produces an interference pattern with a pitch of ∼300 nm, which is set by the wavelength of the laser and the incidence angle.

### 2.2 Growth of DE GaAs/AlGaAs QDs and QRs

The samples were grown on 2-inch epi-ready (100) GaAs wafers by solid source MBE. Prior to the growth, surface native oxides from the GaAs substrates were removed at a substrate temperature (*T*_s_) of about 630 °C under As_2_ supply, after which a 300 nm thick GaAs buffer and a 100 nm Al_0.3_Ga_0.7_As barrier were grown at *T*_s_ = 630 °C. For the formation of Ga droplets, *T*_s_ was lowered to 100 °C and the arsenic valve was closed until the background pressure inside the chamber was decreased to below 3 × 10^−10^ mbar. We estimate that this low pressure is a requirement to prevent residual arsenic from the chamber interacting with the subsequent Ga deposition. Subsequently, *in situ* single pulse DLIP with laser fluence in the range of 40–50 mJ/cm^2^ was introduced on the AlGaAs surface, and the substrate rotation was stopped during the patterning. After a growth interruption of 20 s, a Ga amount equivalent to 2.0–2.5 monolayers (ML) was supplied at a growth rate (GR) of 0.25 ML s^−1^ and resulted in the formation of Ga droplets. Following the Ga deposition, the droplets were subsequently crystallised into GaAs nanocrystals (QDs) under an As_4_ flux at *T*_s_ = 200 °C for 5 min. For the photoluminescence (PL) measurement, the QDs were annealed at a *T*_s_ of 400 °C for 10 min under an As_4_ flux to improve the crystalline quality and then covered with a 10 nm Al_0.3_Ga_0.7_As capping layer which should be sufficient to planarize the surface and prevent the dissolution the GaAs DE quantum dots, otherwise at high temperatures these may transition to two-dimensional (2-D) GaAs nanocrystals. After this initial low-temperature capping, the *T*_s_ was raised to a high temperature of 630 °C and an additional 90 nm Al_0.3_Ga_0.7_As barrier and a final 10 nm GaAs capping layer were grown. After the entire growth, thermal annealing of the entire structure was performed at *T*_s_ = 750 °C for 30 min with an arsenic flux to improve the optical quality. Uncapped samples were also prepared and surface structural characterisation of these was undertaken by atomic force microscopy (AFM).

### 2.3 Growth of SK InAs/GaAs QDs

After the oxide removal, a 500 nm GaAs buffer was grown at *T*_s_ = 600 °C at a GR of 3.0 Å s^−1^, and then *T*_s_ was cooled to 500 °C for InAs QD growth. 1 ML InAs was then supplied at a GR of 0.026 ML s^−1^, and immediately *in situ* single pulse DLIP was applied onto the surface. Laser fluences in the range of 12–25 mJ/cm^2^ were typically utilised for the patterning. Subsequently, further deposition of InAs was supplied to form SK QDs. During DLIP, the substrate rotation was momentarily stopped at the indexing position, and the growth of InAs was not interrupted. For optical characterisation, a double-layer structure that consists of two layers of InAs QDs was fabricated. In this structure, an additional 300 nm layer of AlGaAs layer was grown before the GaAs buffer to enhance the PL signal, and after the first layer of QD growth, a 200 nm GaAs spacer was deposited. After 10 s of growth interruption, the uncapped top layer of InAs QDs guided by DLIP was grown for structural characterisation, which is under identical laser and growth conditions as the buried layer. The surface morphologies of the samples were characterised by AFM.

## 3 Results and discussion

### 3.1 GaAs/AlGaAs QD and QR arrays

**Figure 2:**
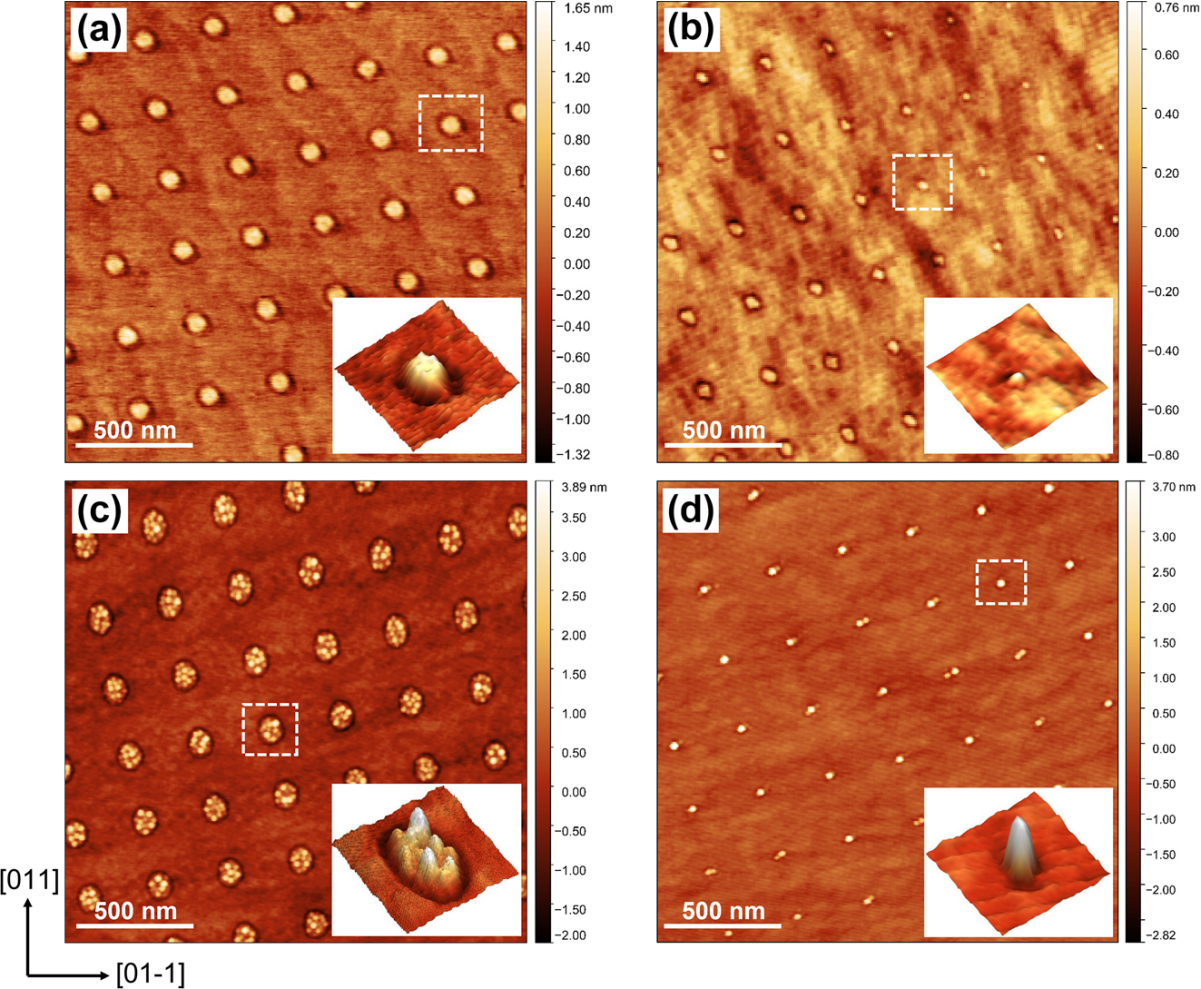
AFM micrographs of (a) and (b) square arrays of nanoislands with different sizes on AlGaAs surface induced by *in situ* four-beam DLIP. 2.5 ML Ga were deposited on the nanoisland-templated surface at *T*_s_ = 100 °C with (c) multiple Ga droplets nucleate at large nanoislands, and (d) single or pair Ga droplets nucleate at small nanoislands. The insets display the enlarged 3-D AFM images.

Square arrays of nanoislands with a pitch of ∼300 nm were observed on the AlGaAs surface after single pulse DLIP, as shown in Figure 2(a) and (b), in which the size difference between these images is due to the spatial variation of the laser intensity which results from the overlap of Gaussian beam profiles. The nanoislands in Figure 2(a) are larger where the laser energy is higher, with a typical height of 1 nm, whilst the islands in Figure 2(b) are relatively small in a region where the laser intensity is lower, with a typical height of 0.5 nm. The smallest islands are approximately 0.3 nm (∼1 ML) high and 20 nm wide. The formation of these nanoisland arrays may be the result of surface migration of adatoms driven by the thermocapillary effect due to 2-D temperature transients generated on the surface [23, 25, 26], or to the Marangoni effect [27–30] where the surface material may be locally melted in the region of the peak intensity interference maxima, perhaps to a depth of only a few monolayers, after which the molten material can flow inward towards the centre induced by the chemicapillary effect. By supplying Ga onto this surface, Ga metallic droplets were generally formed on the islands. Figure 2(c) and (d) show the AFM micrographs of 2.5 ML Ga deposited onto the patterned surface, for which multiple droplets nucleate on relatively large nanoislands, and single or pair droplets can be formed on extremely small islands. In Figure 2(c), high-density Ga droplets nucleate on the islands with an average occupancy of ∼7 droplets per island site. These exhibit a relatively large size fluctuation ranging from 2–4 nm in height. For comparison, it is hard to observe the underlying small islands in Figure 2(d), and 1 to 2 Ga droplet occupancy is attained. The typical height of droplets in this case is ∼4 nm and they show better size uniformity than that on larger islands. In both cases, the nucleation of Ga droplets on the planar area between the nanoislands is suppressed. The results indicate that excellent control of droplet nucleation can be obtained by introducing nanoisland sites on the surface, where the capillary-driven Ga diffusion is greatly enhanced by the presence of the nanoislands, and very small islands around 1–2 ML high are sufficient to drive preferential nucleation.

**Figure 3:**
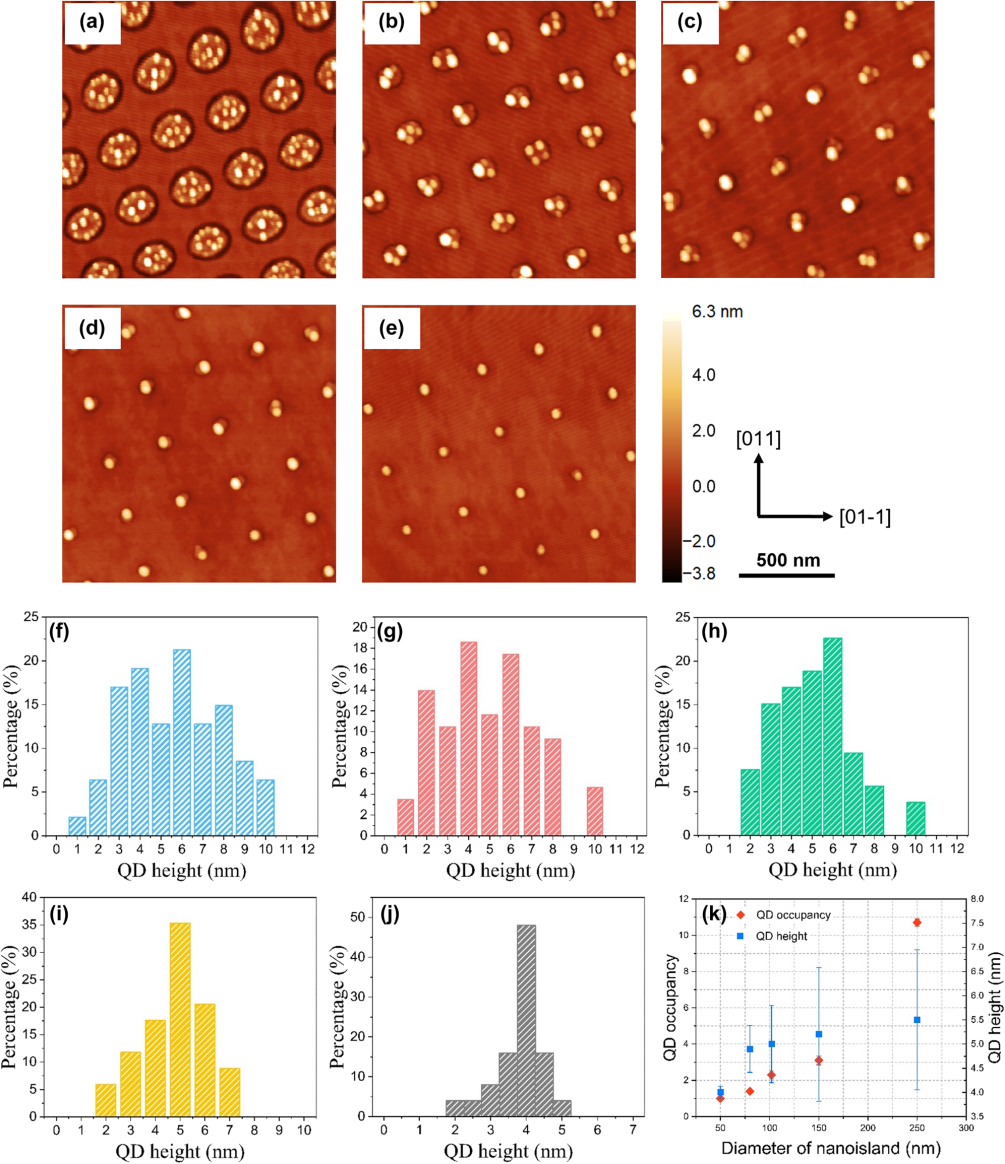
AFM images of crystallised 2 ML GaAs QDs grown at nanoisland-templated surfaces with different diameters of nanoislands (a)–(e) 250, 150, 100, 80 and 50 nm accordingly. (f)–(j) Corresponding histograms of QD height distribution. (k) QD occupancy per nanoisland and QD height in response to the diameter of the nanoisland.

Ga droplets formed on the AlGaAs surface were subsequently crystallised into GaAs QDs under an arsenic flux. The position of GaAs QDs is predominantly dictated by the position of Ga droplets, which have been manifested by the formation of DLIP-induced nanoislands. Figure 3 presents the surface morphologies for 2 ML equivalent Ga droplets after the crystallisation and with a beam equivalent pressure (BEP) As_4_ flux of ∼2.4 × 10^−4^ mbar and *T*_s_ = 200 °C at the crystallisation stage. It is observed that the surface density of the GaAs QDs is proportional to the size of nanoislands, which is comparable to that of initial Ga droplets. Many QDs (>10) were formed on larger nanoislands as shown in Figure 3(a), whereas with a reduction of nanoisland size from 250 nm to 50 nm (a)–(e), the QD occupancy per site also decreases. In Figure 3(e), we observed a well-ordered array of single GaAs QDs on the surface. Figure 3(f)–(j) reveals the height distribution of these GaAs QDs and Figure 3(k) shows the dependence of the dot occupancy and dot height on the diameter of the nanoisland. Large nanoislands are able to accommodate many QDs (3–5 nm high), and due to the coalescence of these QDs, large dots or clusters can be observed with a height of ∼10 nm. Thus, larger nanoislands result in higher QD occupancy and a broader size distribution. As the island size reduces to below 80 nm, only one or two QDs with a dominant height of 4–5 nm were formed and this exhibits a relatively narrow QD height distribution. Large ∼10 nm high QDs were not observed on these small nanoislands. It appears that larger nanoislands are able to attract more Ga atoms to nucleate droplets and this effect weakens as the island size reduces. Similar behaviour can be found in the site-controlled growth of QDs on hole-patterned substrates [31, 32]. We noted that there is no observation of QDs outside the pattern area, implying that the deposition of 2 ML Ga on the planar surface is insufficient for QD formation. However, in the laser-patterned region, the critical thickness for QD formation seems to be locally reduced and thereby we achieve QD growth mainly on the pattern.

**Figure 4:**
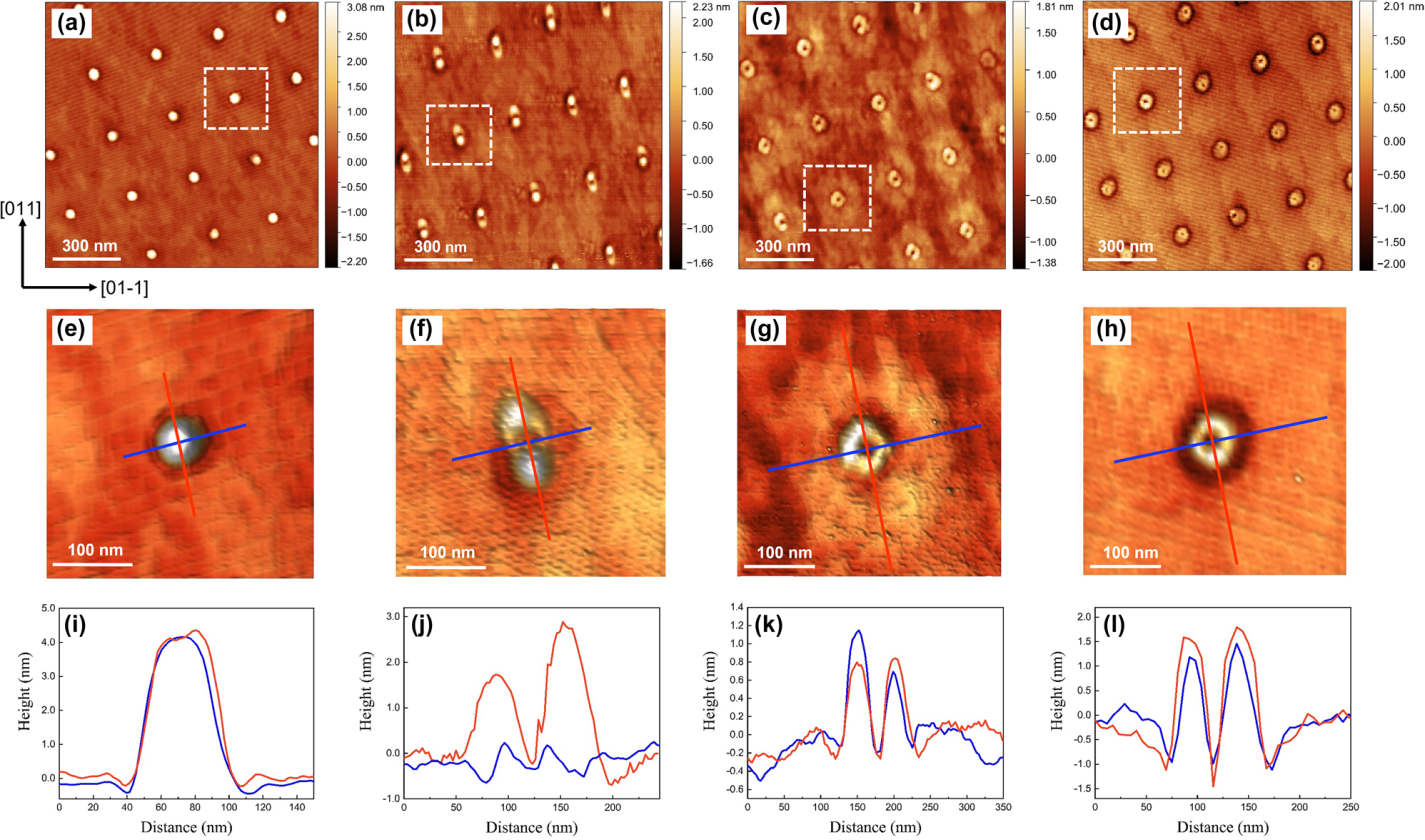
AFM micrographs of 2 ML GaAs QD nanostructure arrays grown at different *T*_s_ and As BEPs for crystallisation of (a) *T*_s_ = 200 °C, BEP = 2.4 × 10^−4^ mbar, (b) *T*_s_ = 400 °C, BEP = 1 × 10^−4^ mbar, (c) *T*_s_ = 400 °C, BEP = 2.3 × 10^−5^ mbar, and (d) *T*_s_ = 400 °C, BEP = 1.4 × 10^−6^ mbar. (e)–(h) The corresponding enlarged AFM images of single QD structures as marked in (a)–(d), respectively. (i)–(l) Line scans of each QD structure.

The arsenic (As) flux during the crystallisation is of critical importance in controlling the shape of QD structures grown using the DE method. As well as QDs, it has been demonstrated that it is possible to fabricate single QRs, double QRs or even multiple rings by carefully tuning the As flux during the droplet crystallisation [33–36]. Figure 4 reveals AFM micrographs of four GaAs QD sample surfaces with the same 2 ML equivalent Ga deposition at 100 °C and subsequently crystallised by As_4_ without annealing_,_ but with different crystallisation temperatures of (a) 200 °C, (b–d) 400 °C and reducing As BEPs: (a) 2.4 × 10^−4^ mbar, (b) 1 × 10^−4^ mbar, (c) 2.3 × 10^−5^ mbar and (d) 1.4 × 10^−6^ mbar. In terms of a higher As BEP as shown in Figure 4(a), an array of single GaAs QDs with a typical height of ∼4 nm was formed. The dot-like shape is comparable with that of the original Ga droplet. When the As BEP was decreased to 1 × 10^−4^ mbar, the shape of GaAs nanostructures was transformed to elongated rings, in which two QDs were laterally coupled, as displayed in Figure 4(b). These asymmetric rings have a height of around 2–3 nm and are separated by a distance of ∼15 nm. With a further decrease in the arsenic BEP, these asymmetric GaAs QRs were transformed to symmetric QRs which contain quasi-2-D disks in their outer region as shown in Figure 4(c). The ring disks are 300 nm wide and 1 ML high on average, and the inner rings are ∼1 nm high and with a width of 60–80 nm. The base size of the inner rings is similar to that of the QDs shown in Figure 4(a), which corresponds to the size of the original Ga droplets. At an even lower As BEP of 1.4 × 10^−6^ mbar as displayed in Figure 4(d), symmetric QRs without disks were formed. The size of these rings is similar to that of the inner rings in (c), which is associated with the original droplet size. The enlarged AFM images of each representative structure are shown in Figure 4(e)–(h), and the corresponding cross-sectional profiles along the directions marked as red and blue lines are presented in Figure 4(i)–(l), respectively. It is noticeable that the height of GaAs structures also decreases with the reduction in the As flux BEP.

The shape evolution of GaAs nanostructures is ascribed to the competition between different incorporation mechanisms during As flux irradiation [34, 37], [38], [39]. It was reported that the inner QR structure is already formed at the edge of the droplet just after Ga droplet formation, due to residual As atoms from the underlying substrate or the chamber [34]. These As atoms can become dissolved beneath the droplets and then diffuse to the droplet edge driven by an internal convection flux. Thus, the size of the inner rings is consistent with that of the original Ga droplets. The final shape of the GaAs nanostructures is governed by the counter-diffusion process of Ga atom diffusion from the droplets and As atom diffusion towards the droplets [33]. When an As impinging flux is supplied onto the surface, atoms from the droplets are able to diffuse towards the As-stabilised surfaces and result in crystallisation at a relatively large distance from the Ga droplets [34]. Through such a mechanism the outer disks can be formed. The diameter of the outer disk is controlled by the Ga diffusion length [37]. By either increasing the *T*_s_ or reducing the As BEP, it is possible to increase the diameter of the outer disk. The 2-D growth of GaAs thin layers is expected in the case of an extremely low arsenic flux. Generally, resulting from the shape of the Ga droplet, the nanostructure formed at a low As BEP is reasonably isotropic. At a relatively high As BEP, elongated structures can be formed by virtue of the anisotropic diffusion length of Ga atoms [40]. With a further increase in the As flux intensity, the Ga diffusion is restrained whilst crystallisation is favoured, which enhances the three-dimensional (3-D) QD growth.

**Figure 5:**
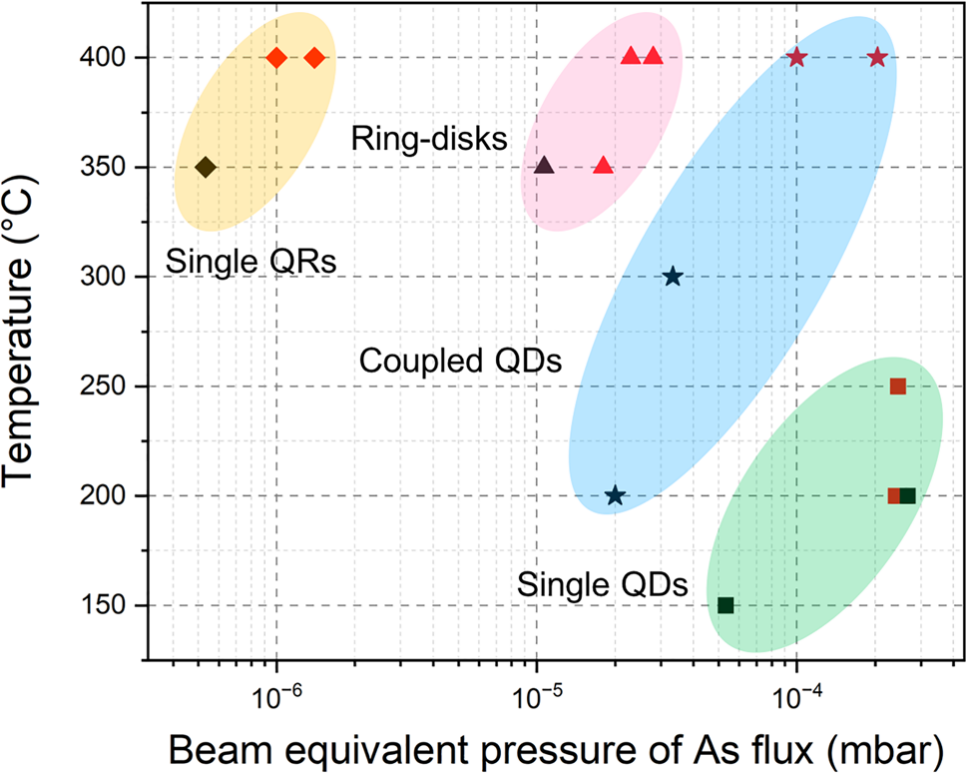
Graph of various structures formed at different crystallisation temperatures and As BEPs. ◆: single rings, ▲: ring-disks, ★: coupled QDs, ■: single QDs. Red symbols represent our experimental data, and black symbols refer to experimental data from other reported work [33, 35, 41, 42, 43].

With different growth parameters, we are able to fabricate a variety of GaAs QD structure arrays, ranging from single QDs, coupled QDs to single QRs. Figure 5 summarises the formation of various nanostructures at different temperatures during the crystallisation and BEPs of As flux based on our experimental data and other reported results [33, 35, 41, 42, 43]. For the fabrication of single rings, relatively high crystallisation temperatures and low As BEPs are preferable. In contrast, lower temperatures and higher As BEPs are essential for the formation of single QDs.

**Figure 6:**
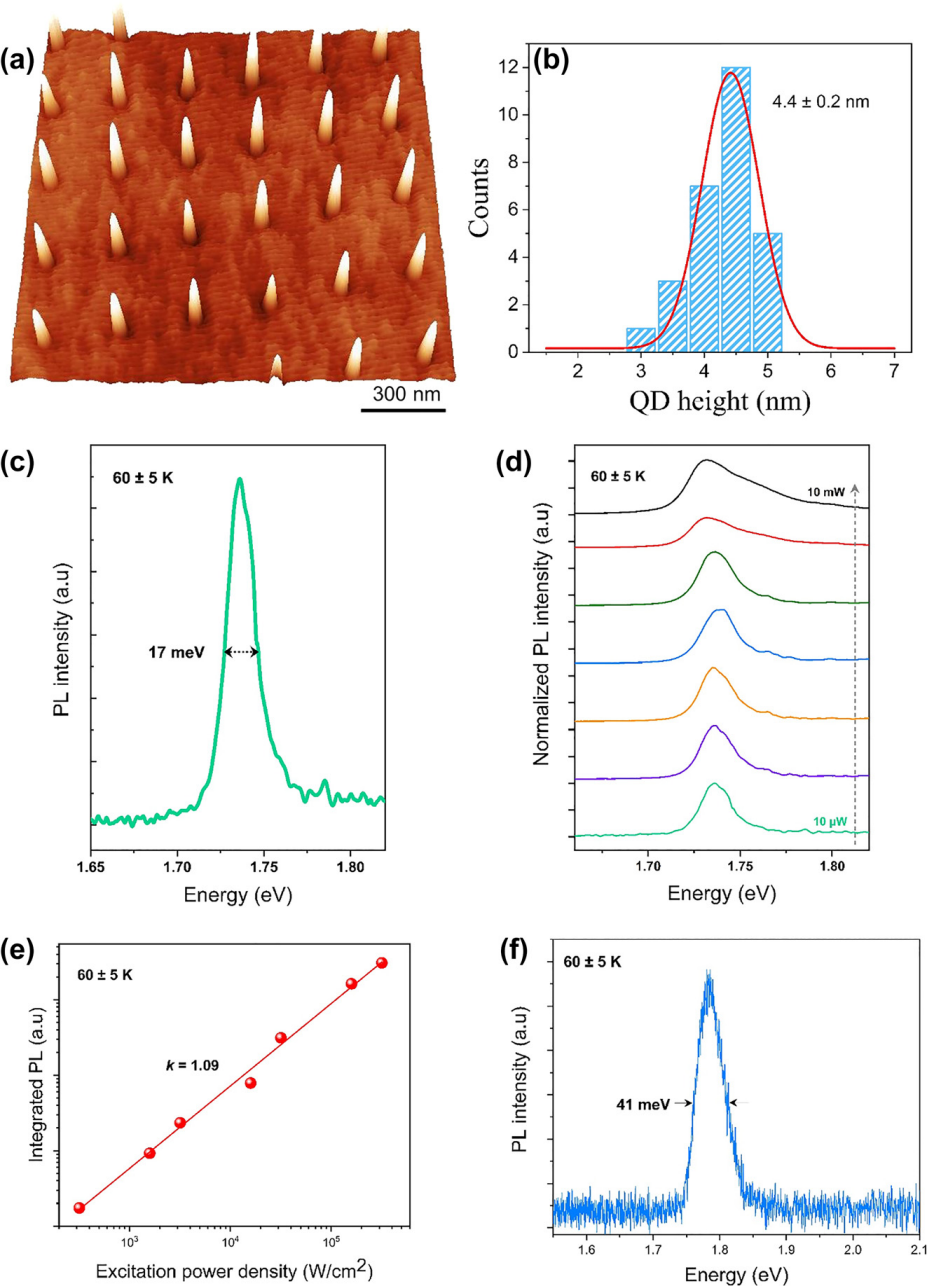
Characterisation of patterned GaAs QD structures. (a) 3-D AFM micrograph of a regular array of single GaAs/AlGaAs QDs. (b) Corresponding QD height histogram. (c) The ensemble-PL spectrum of the patterned GaAs QD arrays with low excitation power at 60 ± 5 K. (d) Normalised excitation power-dependent PL spectra. (e) Integrated PL intensity depending upon the excitation power density. The solid line defines the slope *k =* 1.09*.* (f) PL spectrum of the patterned GaAs QR arrays with an excitation power of 10 µW.

Figure 6(a) presents a 3-D AFM micrograph of a uniform array of single GaAs QDs, with the dot height histogram displayed in (b). Areas of the wafer outside of the laser spot do not show the formation of QDs and instead only 2-D monolayer GaAs terraces appear. The optical properties of the fabricated GaAs QD arrays and QR arrays were characterised by low-temperature PL measurements. The PL measurement of the samples was carried out at a closed cycle cryostat and excited utilising a 594 nm laser, and a 100× objective was used to collect the PL. The laser spot size in these conditions is 2–3 μm. The actual sample temperature was measured at around 60 K derived from the GaAs band edge emission. Figure 6(c) manifests the PL spectrum of the emission from an ensemble of patterned GaAs QD arrays at low excitation power and (d) depicts the normalised excitation-power-dependent PL spectra with an increment of excitation power from 10 µW to 10 mW.

A 1.74 eV PL emission peak with a record narrow full width at half maxima (FWHM) of ∼17 meV was observed at low excitation power. At relatively low excitation power, there is no considerable alteration in the shape of the spectrum or shift of the peak energy. The PL peak is slightly redshifted by 5 meV at higher excitation power of 5–10 mW. The redshift might result from the effect of band-gap re-normalisation where the band gap shrinks with increasing carrier density due to Coulomb interaction [44, 45], or it could be attributed to the thermal effects occurring in a closed cycle cryostat at high excitation power density. The separation of the ground and excited states in this QD system is too small to be resolved. Figure 6(e) presents the integrated PL intensity of the patterned GaAs QDs as a function of the excitation power density at 60 K. The slope *k* ≈ 1 reveals a linear dependence, suggesting excitonic recombination is dominant. Figure 6(f) exhibits the PL spectrum of the patterned GaAs QR arrays displayed in Figure 4(c), regarding the low excitation power of 10 µW, the PL peak emission was at 1.78 eV, and the FWHM is approximately 41 meV. Compared with the GaAs QDs, QRs have a smaller dot size of ∼1 nm in height, giving rise to larger emission energy, and the relatively broader linewidth results from greater size inhomogeneity.

### 3.2 InAs/GaAs QD arrays

**Figure 7:**
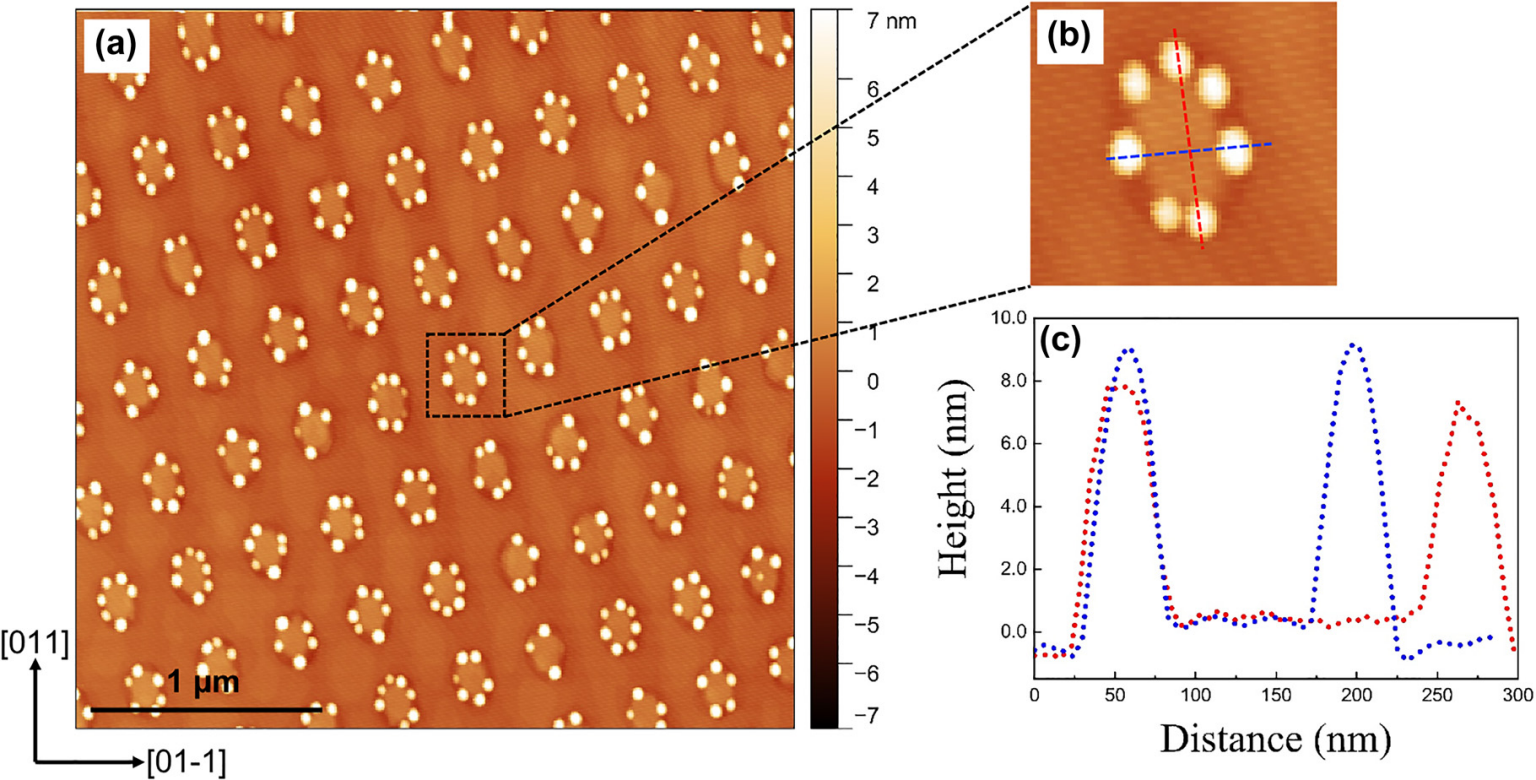
AFM micrographs of (a) InAs QDs formed on the nanoisland-templated surface with a total InAs coverage of 1.7 ML, and (b) the magnified AFM image of a single nanoisland site. (c) Line scans across the direction as marked in (b).

Similar to the patterning of GaAs/AlGaAs QDs, after the initial deposition of 1 ML InAs, single pulse four-beam DLIP with a laser fluence of approximately 15 mJ/cm^2^ was applied *in situ* on the sample surface. The 1 ML thickness of InAs was selected as it is below the critical thickness, and it would be expected to remain at this stage a relatively smooth surface characterised by monolayer terraces. After the pulse, a square array of nanoislands was produced on the surface, with the same period of 300 nm. Further deposition of InAs leads to the formation of SK InAs QDs at the nanoisland sites, as shown in Figure 7.

**Figure 8:**
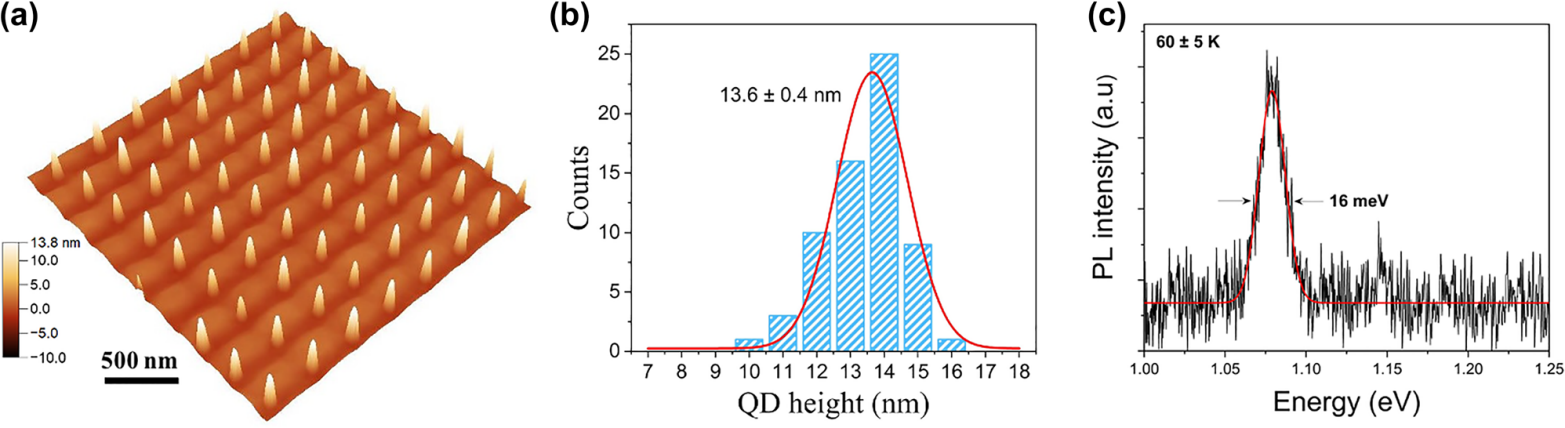
Characterisation of patterned InAs QDs. (a) 3-D AFM micrograph of an array of single InAs/GaAs QDs induced by *in situ* DLIP. (b) Histogram of QD height distributions. (c) Low-temperature PL spectrum with an excitation power of 1.5 µW.

It is observed that InAs QDs are preferentially nucleated at the edge of the nanoislands and the nucleation of interstitial QDs on the planar areas between the island sites is completely suppressed. The size distribution of these nucleated QDs exhibits a large fluctuation varying from 2 to 10 nm in height. The typical size is ∼50 nm wide and ∼8 nm high, whereas ∼20 nm wide and ∼several MLs high small QDs are also observed. The nucleation sites of QDs at the edge of islands and the occupancy of QDs at each island site are randomly distributed, since the edges of these islands still present large areas for nucleation. In expectation of attaining uniform dot occupancy and size distribution, the growth conditions as well as the nanoisland size need to be optimised. A detailed study of the effect of growth and DLIP parameters on the formation of InAs QDs can be found in our previous work [46]. To achieve uniform single InAs QD arrays, a subcritical InAs coverage of ∼1.5 ML, a low GR of 0.026 ML s^−1^ and the formation of small nanoislands due to low laser intensity are essential. Figure 8(a) presents an AFM image of a regular array of fabricated single SK InAs QDs on the GaAs surface and (b) depicts the QD height histogram.

PL measurements of the fabricated InAs QD arrays were performed at 60 K using a 594 nm laser and a 100× objective. The PL signal was spectrally resolved by a spectrometer and a liquid nitrogen cooled InGaAs detector. Figure 8(c) presents an ensemble-PL spectrum from the InAs QD array with an excitation power of 1.5 µW. The QD emission is centred at 1.08 eV and a very narrow FWHM of 16 meV is observed, which is smaller than most values of the reported site-controlled InAs QDs [13, 31, 47, 48]. This result confirms the good optical quality of these patterned InAs QDs and indicates that the size variation in these QDs is reasonably small.

Our results of DE and SK QD growth exhibit qualitatively similar behaviour, that the formation of nanoislands on the underlying planar surface by *in situ* DLIP is critical to control the nucleation of QDs and droplets. DLIP is able to modify both InGaAs and AlGaAs surfaces, albeit requiring slightly higher laser energy for the AlGaAs case due to larger activation energy for diffusion and higher reflection coefficient for the UV laser light. In the case of the DE method, Ga droplets are nucleated on the nanoislands as a result of enhanced Ga adatom diffusion towards the islands. To form droplets in Ga-rich areas, we must have an order of magnitude similar diffusion rates for Ga adatoms when unaccompanied by arsenic. Note that a very low arsenic system pressure is essential to achieve DE growth. Regarding the SK growth of InAs/GaAs QDs, indium adatom diffusion on an arsenic-terminated GaAs surface is mainly responsible for dot nucleation, and InAs QDs are preferentially formed at the edges of the nanoislands rather than atop the islands. To achieve homogeneous arrays of DE and SK QDs with single dot occupancy, the presence of small nanoislands and optimised growth conditions are of significance.

## 4 Conclusions

We have presented an innovative fabrication route for the realisation of high-quality ordered semiconductor QD and QR arrays by combining the MBE self-assembly (DE and SK growth) with *in situ* direct laser interference patterning (DLIP). In this approach, UV nanosecond single pulse DLIP was applied to a high vacuum MBE system via optical viewports to directly pattern the surface of samples during epitaxial growth. The results show that the applied interference pattern induces the initial formation of periodic arrays of monolayer-high nanoislands with a pitch of 300 nm on the surface. These islands yield energetically preferential nucleation sites for the self-assembly of QDs or droplets. Highly ordered arrays of DE grown single GaAs QDs and QRs on AlGaAs surfaces, and SK grown InAs QDs on GaAs surfaces have been obtained through careful control of the pulse energy and the epitaxial growth parameters. Such samples exhibit high optical quality and uniformity with record narrow photoluminescence linewidths. Compared to traditional pattern-etch-regrowth processes, the use of DLIP offers a fast, single-step and high-throughput technique for patterned self-assembly. Further refinement of the technique can create an appealing platform in the fabrication of semiconductor QD arrays for applications in quantum technologies. The resulting QD/QR arrays can provide an ideal basis for the realisation of deterministic quantum nanophotonics.
